# Author Correction: Stem cell-derived synthetic embryos self-assemble by exploiting cadherin codes and cortical tension

**DOI:** 10.1038/s41556-023-01157-1

**Published:** 2023-05-23

**Authors:** Min Bao, Jake Cornwall-Scoones, Estefania Sanchez-Vasquez, Andy L. Cox, Dong-Yuan Chen, Joachim De Jonghe, Shahriar Shadkhoo, Florian Hollfelder, Matt Thomson, David M. Glover, Magdalena Zernicka-Goetz

**Affiliations:** 1 Division of Biology and Biological Engineering, California Institute of Technology, Pasadena, CA USA; 2 Mammalian Embryo and Stem Cell Group, Department of Physiology, Development and Neuroscience, University of Cambridge, Cambridge, UK; 3 The Francis Crick Institute, London, UK; 4 Department of Biochemistry, University of Cambridge, Cambridge, UK

**Keywords:** Embryonic stem cells, Stem-cell biotechnology, Embryology

Correction to: *Nature Cell Biology* 10.1038/s41556-022-00984-y. Published online 13 September 2022.

In the version of this article initially published, author Andy L. Cox (Division of Biology and Biological Engineering, California Institute of Technology, Pasadena, CA, USA) was mistakenly omitted from the author list. The error has been corrected in the HTML and PDF versions of the article.

