# Supplementary material for: Stem cell-derived synthetic embryos self-assemble by exploiting cadherin codes and cortical tension
Source: Nat Cell Biol. 2022 Sep 13;24(9):1341–9. doi: 10.1038/s41556-022-00984-y (PMC9481465; doi:10.1038/s41556-022-00984-y)
Supplement: Supplementary file 1 — Supplementary information for modelling. [file 41556_2022_984_MOESM1_ESM.pdf]

nature portfolio

https://doi.org/10.1038/s41556-022-00984-y

Supplementary information

Stem cell-derived synthetic embryos self-assemble by exploiting cadherin codes and cortical tension

In the format provided by the authors and unedited

Supplementary Information

Stem cell derived synthetic embryos self-assemble by exploiting cadherin codes and cortical tension

Min Bao[1,2], Jake Cornwall-Scoones[1,3], Estefania Sanchez-Vasquez[1], Dong-Yuan Chen[1], Joachim De Jonghe[3], Shahriar Shadkhoo[1], Florian Hollfelder[4], Matt Thomson[1], David M. Glover[1], and Magdalena Zernicka-Goetz[1,2] *

[1] California Institute of Technology, Division of Biology and Biological Engineering, 1200 E. California Boulevard, Pasadena, CA 91125, USA

[2] Mammalian Embryo and Stem Cell Group, Department of Physiology, Development, and Neuroscience, University of Cambridge, Cambridge CB2 3DY, UK

[3] Present address: The Francis Crick Institute, London NW1 1AT, UK

[4] Department of Biochemistry, University of Cambridge, Cambridge CB2 1GA, UK

* Corresponding author: mz205@cam.ac.uk

Supplementary modelling

A Cellular Potts Model (CPM)[1] was used to infer the predicted distributions of conformations given measurements of cell adhesion from AFM, and to determine the roles of cortical stiffness on self-organization of ETX-embryos.

Model objects

Cells occupy contiguous sets of points in a square lattice of size $(N_x \times N_y)$. Each cell is prescribed a unique id, recorded in matrix $\mathbf{I}$ $(N_x \times N_y)$. Further, each cell is prescribed a cell type (e.g. ES, TS, XEN), entailing unique, pre-defined cellular properties. Cell type is immutable, establishing a mapping between the cell index i and its cell type $c_i = 2$. Lattice points that are unoccupied by a cell define the medium, given an id $i = 0$ and $c_0 = 0$.

Energy functional

The simulation evolves via a stochastic minimization of an energy function that accounts for both differential affinity and other physical properties of cells. The energy functional was defined as below:

$$E = \sum_{i=1}^{n_c} \underbrace{\lambda_{A,i}(A_i - A_{i,0})^2}_{Area\ penalty} + \underbrace{\lambda_{P,i}(P_i^2 + \kappa b_i)}_{Contractility} + \underbrace{\lambda_T \sum_{x,y \in \omega_i} \sum_{dx,dy \in \Omega} J_{i,I_{x+dx,y+dy}}}_{Adhesion\ /\ Tension} \quad \text{(eq. 1)}$$

$\lambda_{A,i}$ describes the bulk modulus of area deformations of a cell i from its optimum $A_{i,0}$. $\lambda_{P,i}$ defines its circumferential elastic modulus of the perimeter, scaling a contractility term (P_i^2) and the tension of interfaces between cells and the media (κb_i where b_i is the number of Moore neighbors of cell i that are medium). The final term accounts for adhesion/tension with neighboring cells: ω_i is the set of lattice points x, y that the cell occupies; Ω is the Moore neighborhood; meaning $I_{x+dx,y+dy}$ is the cell id of a lattice point that neighbors a point within the cell; $J_{i,I_{x+dx,y+dy}}$ defines the strength of the interaction between cell i and the neighboring cell; and λ_T is a scale-factor across all adhesion terms. $\mathbf{J}$ is a symmetric matrix $(n_c + 1 \times n_c + 1)$ of pairwise interaction strengths. Interactions must be between different cells, meaning $J_{ii} = 0\ \forall i$.

The matrix $\mathbf{I}$ defines the area and perimeters of each cell. The area A_i of cell i is defined as the number of lattice point that cell i occupies, i.e.:

$$A_i = \sum_{x=1}^{N_x} \sum_{y=1}^{N_y} \delta_{i,I_{x,y}} \quad \text{(eq. 2)}$$

Likewise, the perimeter P_i of cell i is the number of lattice points that are: (i) members of the Moore neighborhood of the lattice points of cell i (i.e. ω_i); but (ii) are not themselves members of the cell i.

Bootstrapping procedure

We parameterized adhesion strengths using cohesion forces between pairs of cell-types that were directly measured by AFM. For each simulation, we sampled this distribution to build the $\mathbf{J}$ matrix. Specifically, for a given element J_{ij} we sample (with replacement) the set of AFM cohesion forces measured between cell-types c_i and c_j (e.g. ES-ES, ES-TS,…), while enforcing symmetry in the $\mathbf{J}$ matrix. We set entries between cells and the medium (J_{0j}, J_{i0}) to 0. Bootstrap sampling is performed around 500 times to establish an ensemble of $\mathbf{J}$ matrix samples. Each $\mathbf{J}$ matrix sample is used to perform a CPM simulation, generating an ensemble distribution of conformations over time.

Simulation algorithm

The CPM evolves via a stochastic minimization. In each Markov Chain Step (MCS), a random lattice site is selected. One of the four sites in the Von Neumann neighborhood is then selected and the state of the chosen site is putatively reassigned to that of its neighbor. The energy functional is then evaluated before and after the swap, defining ΔE. The swap is then accepted only if:

$$\Delta E = \min(1, exp(\frac{-\Delta E}{T})) \quad \text{(eq. 3)}$$

As with the lattice model, T defines the effective temperature of the system, modulating the propensity to perform energetically unfavorable swaps. In traditional CPM simulations, cell Moore contiguity breaks down at high T given swapping rules are

local. Consequently, we universally reject potential state changes that compromise contiguity [2].

Automated scoring of conformations

To determine the conformation of a simulated structure at a given time-point, we established an automated scoring procedure. Firstly, we remove cells that have detached from the main aggregate by calculating the adjacency matrix between cells (Moore neighborhood) and removing all clusters besides the one with the largest number of connected components. Secondly, we score each cell-type for envelopment. A cell-type is defined to be enveloping if its center of mass lies within a different cell-type, rather than that of its own. Thirdly, we score cell-type contiguity by calculating the subgraph of the connectivity matrix that contains only cells of a given type, then determining whether the number of connected components is 1 (i.e. contiguous). With three cell-types, there are 16 possible completely sorted conformations. These conformations can be divided into 4 categories.

In category (1) conformations, two cell types sequentially envelope a third. The order of envelopment is determined via adjacency among cell-types. For example, when E envelopes X which envelopes T: at least one X must contact T; at least one E must contact the medium; at least one E must contact X; and no E should contact T. Further, the inner most cell-type must be contiguous.

In category (2), one cell type envelopes another, with a third attached peripherally; whereas in category (3) one cell type envelopes the other two (as in ETX embryos). Both categories must contain two contiguous cell-types and a third enveloping cell-type. If all cells of the enveloping cell-type contact the medium, the conformation is scored to category (3). If any of the cells that do not contact the medium are instead surrounded by a single cell-type, the conformation is scored as ‘unsorted’. Alternatively, if any of these cells contact exactly two other cell-types, then the conformation falls in category (2). Which variant within category (2) is determined by counting the number of contacts (e.g. X envelopes E rather than T if X and E share more contacts than X and T). Otherwise, the conformation is assigned category (3).

Category (4) is assigned when all three cell-types are non-enveloping and are contiguous. If a given structure does not fall within any of these categories, it is classed as ‘unsorted’.

Additionally, we define cell externalization: if all cells of that type either contact the medium directly, or are connected to cells that are connected to the medium. Strictly, we define the subgraph of the adjacency matrix containing the rows and columns of a given cell-type plus the medium; if this subgraph has a single connected component, then the cell-type is externalized.

Lower stiffness in XEN cells improves the speed and fidelity of their externalization

We used the CPM to determine whether reduced stiffness in XEN cells can explain the robustness of their externalization *in silico*. We systematically altered the stiffness of XEN cells by varying the circumferential elastic modulus of XEN cells λ_P^{XEN} between 0.04 and 0.20 (9 values simulated). This parameter ascribes the extent of the circumferential energy penalty, meaning a cell with a higher values of λ_P^{XEN} resists deformations to its perimeter more i.e. is stiffer.

1 Graner, F. & Glazier, J. A. Simulation of biological cell sorting using a two-dimensional extended Potts model. *Physical review letters* **69**, 2013 (1992).
2 Durand, M. & Guesnet, E. An efficient Cellular Potts Model algorithm that forbids cell fragmentation. *Computer Physics Communications* **208**, 54-63 (2016).
