# Supplementary material for: Stem cell-derived synthetic embryos self-assemble by exploiting cadherin codes and cortical tension
Source: Nat Cell Biol. 2022 Sep 13;24(9):1341–9. doi: 10.1038/s41556-022-00984-y (PMC9481465; doi:10.1038/s41556-022-00984-y)
Supplement: Supplementary file 2 — Reporting Summary [file 41556_2022_984_MOESM2_ESM.pdf]

nature portfolio

Corresponding author(s): Magdalena Zernicka-Goetz

Last updated by author(s): Jun 17, 2022

Reporting Summary

Nature Portfolio wishes to improve the reproducibility of the work that we publish. This form provides structure for consistency and transparency in reporting. For further information on Nature Portfolio policies, see our Editorial Policies and the Editorial Policy Checklist.

Statistics

For all statistical analyses, confirm that the following items are present in the figure legend, table legend, main text, or Methods section.

n/a	Confirmed	
☐	☒	The exact sample size (*n*) for each experimental group/condition, given as a discrete number and unit of measurement
☐	☒	A statement on whether measurements were taken from distinct samples or whether the same sample was measured repeatedly
☐	☒	The statistical test(s) used AND whether they are one- or two-sided<br>*Only common tests should be described solely by name; describe more complex techniques in the Methods section.*
☐	☒	A description of all covariates tested
☐	☒	A description of any assumptions or corrections, such as tests of normality and adjustment for multiple comparisons
☐	☒	A full description of the statistical parameters including central tendency (e.g. means) or other basic estimates (e.g. regression coefficient) AND variation (e.g. standard deviation) or associated estimates of uncertainty (e.g. confidence intervals)
☐	☒	For null hypothesis testing, the test statistic (e.g. F, t, r) with confidence intervals, effect sizes, degrees of freedom and P value noted<br>*Give P values as exact values whenever suitable.*
☒	☐	For Bayesian analysis, information on the choice of priors and Markov chain Monte Carlo settings
☒	☐	For hierarchical and complex designs, identification of the appropriate level for tests and full reporting of outcomes
☒	☐	Estimates of effect sizes (e.g. Cohen's *d*, Pearson's *r*), indicating how they were calculated

Our web collection on statistics for biologists contains articles on many of the points above.

Software and code

Policy information about availability of computer code

Data collection	LEICA software LAS X was used for image acquisition.
Data analysis	For graphical statistics and statistical tests , GraphPad Prism (V8.0 & 7.0a) was used.<br>Fiji image processing software and Imaris software were used for image processing and analysis.<br>JPK IP Software was used for calculating cell adhesion force and cortical stiffness.<br>NanoScope software (V6.13) was used for sample stage calibration before loading the sample during AFM experiment.<br>FlowJo software (V10.7.1) was used to plot E- and P-cadherin intensity, measured from FACS experiment.

For manuscripts utilizing custom algorithms or software that are central to the research but not yet described in published literature, software must be made available to editors and reviewers. We strongly encourage code deposition in a community repository (e.g. GitHub). See the Nature Portfolio guidelines for submitting code & software for further information.

Data

Policy information about availability of data

All manuscripts must include a data availability statement. This statement should provide the following information, where applicable:

- Accession codes, unique identifiers, or web links for publicly available datasets
- A description of any restrictions on data availability
- For clinical datasets or third party data, please ensure that the statement adheres to our policy

Previously published scRNA-seq data that were re-analysed here are available under accession code GSE161947. Source numerical data are provided with this paper. All relevant data supporting the key findings of this study are available within the article and its Supplementary Information files. All other data supporting the findings of this study are available from the corresponding author on reasonable request.

Field-specific reporting

Please select the one below that is the best fit for your research. If you are not sure, read the appropriate sections before making your selection.

☒ Life sciences ☐ Behavioural & social sciences ☐ Ecological, evolutionary & environmental sciences

For a reference copy of the document with all sections, see nature.com/documents/nr-reporting-summary-flat.pdf

Life sciences study design

All studies must disclose on these points even when the disclosure is negative.

Sample size	Sample size was determined based on our previous experience and the work of other groups using stem cell derived embryos and mouse natural embryos as experimental model systems (Nature cell biology 20.8 (2018): 979-989; Developmental cell 56.3 (2021): 366-382)
Data exclusions	ETX-embryo samples for scRNA-sequencing analysis: those samples that did not pass the quality controls were excluded from the analysis.
Replication	Each result described in the paper is based on at least three independent biological replicates. Figure legends indicate the number of independent experiments performed in each analysis.
Randomization	Samples (mouse embryos) were allocated randomly into experimental groups.<br>The in vitro cell experiments were not randomized as it was not necessary.<br>For experiments with chemical inhibitors, samples were randomly allocated to control and experimental groups.<br>Embryos were randomly allocated to control and experimental groups for in vivo experiments.
Blinding	The investigators were not blinded to group allocation, because this study investigates the fundamental self-organization principles in both stem cell derived and natural embryos, blinding is not relevant to our study and the experiments were descriptive in their nature.

Reporting for specific materials, systems and methods

We require information from authors about some types of materials, experimental systems and methods used in many studies. Here, indicate whether each material, system or method listed is relevant to your study. If you are not sure if a list item applies to your research, read the appropriate section before selecting a response.

Materials & experimental systems

n/a	Involved in the study	
☐	☒	Antibodies
☐	☒	Eukaryotic cell lines
☒	☐	Palaeontology and archaeology
☐	☒	Animals and other organisms
☒	☐	Human research participants
☒	☐	Clinical data
☒	☐	Dual use research of concern

Methods

n/a	Involved in the study	
☒	☐	ChIP-seq
☐	☒	Flow cytometry
☒	☐	MRI-based neuroimaging

Antibodies

Antibodies used

Primary antibodies:
Goat polyclonal anti-Tfap2c R&D Systems Cat# AF5059; RRID: AB_2255891 (1:200)
Goat polyclonal anti-Brachyury R&D Systems Cat# AF2085; RRID: AB_2200235 (1:200)
Rabbit monoclonal anti-Gata4 Cell Signaling Technology Cat# 36966; RRID: AB_2799108 (1:500)
Rabbit polyclonal anti-laminin Sigma-Aldrich Cat# L9393; RRID: AB_477163 (1:500)
Mouse monoclonal anti-Oct4 Santa Cruz Biotechnology Cat# sc-5279; RRID: AB_628051 (1:500)
Rat Monoclonal anti-E-cadherin Thermo Fisher Scientific Cat# 13-1900; RRID: AB_2533005 (1:200)
Goat Monoclonal anti-P-cadherin Santa Cruz Biotechnology Cat# sc-1501; RRID: AB_630961 (1:100)
Mouse Monoclonal anti-P-cadherin Fisher Scientific Cat# MS-1741; RRID: AB_149083 (1:100)

Secondary antibodies:
Donkey anti-Mouse IgG (H+L), Alexa Fluor 488 Thermo Fisher Scientific Cat# A-21202; RRID: AB_141607 (1:500)
Donkey anti-Goat IgG (H+L), Alexa Fluor 488 Thermo Fisher Scientific Cat# A-11055; RRID: AB_2534102 (1:500)
Donkey anti-Rat IgG (H+L), Alexa Fluor 488 Thermo Fisher Scientific Cat# A-21208; RRID: AB_2535794 (1:500)
Donkey anti-Rabbit IgG (H+L), Alexa Fluor 568 Thermo Fisher Scientific Cat# A10042; RRID: AB_2534017 (1:500)
Donkey anti-Mouse IgG (H+L), Alexa Fluor 568 Thermo Fisher Scientific Cat# A10037; RRID: AB_2534013 (1:500)
Donkey anti-Rabbit IgG (H+L), Alexa Fluor 647 Thermo Fisher Scientific Cat# A-31573; RRID: AB_2536183 (1:500)

	Donkey anti-Goat IgG (H+L), Alexa Fluor 647 Thermo Fisher Scientific Cat# A-21447; RRID: AB_2535864 (1:500)<br>Alexa Fluor™ Plus 405 Phalloidin Thermo Fisher Scientific Cat# A30104 (1:200)
Validation	The subcellular localization of all the proteins analyzed in this study has been previously reported.<br>Tfap2c: It correctly stained TS cells and the extra-embryonic ectoderm cells in post-implantation embryos (Science 356, doi:10.1126/ science.aal1810).<br>Brachyury: It correctly stained mesoderm at 6.5 and later as reported and expected (Dev Biol 288, 363-371).<br>Gata4: It correctly labelled XEN cells and the visceral endoderm in postimplantation embryos (eLife 2018;7:e32839)<br>Laminin: It correctly stained the basement membrane between visceral endoderm and Exe or epiblast, as reported elsewhere and as expected (Dev Dyn 241, 270-283)<br>Oct4: It specifically stained ES cells and the epiblast at all stages tested, as expected (Science 356, doi:10.1126/science.aal1810).<br>E-cadherin: It correctly stained the cell-cell junction and basolateral side of cells in the embryo as reported and as expected (Science 356, doi:10.1126/ science.aal1810)<br>P-cadherin: It correctly stained the cell-cell junction as reported (Cell 123.5 (2005): 917-929)<br>F-actin: it correctly stained the cell membrane and was apically enriched, as expected and reported (Development, 138(2011) 3011-3020).

Eukaryotic cell lines

Policy information about cell lines

Cell line source(s)	Experiments were performed using mouse E14 wild-type ES cells (derived in Zernicka-Goetz's lab). Wild-type TS cells (a gift from Jenny Nichols), and wild-type XEN cells (a gift from Ellen Na). Cdh1 and Cdh6 overexpressing ES cells were generated from E14 wild-type ES cells; Cdh3 overexpressing TS cells were generated from wild-type TS cells; Cdh1 and Cdh6 overexpressing XEN cells were generated from wild-type XEN cells (see below). Details can be found in method section in the paper.
Authentication	Cells were maintained in conditions to preserve stem cell character and prevent differentiation. Plates were inspected for morphological evidence of differentiation (altered colony morphology in ESC cultures or presence of trophoblast giant cells in TSC cultures..etc) and plates with differentiated cells were discarded. Furthermore, cell identities were confirmed routinely by immunoflourescence marker expressions.
Mycoplasma contamination	Cell lines were routinely tested for mycoplasma contamination by PCR
Commonly misidentified lines<br>(See ICLAC register)	The cells we used are not part of this database

Animals and other organisms

Policy information about studies involving animals; ARRIVE guidelines recommended for reporting animal research

Laboratory animals	Mice (Mus musculus) were used to obtain mouse embryos for this study. Six-week-old female CD-1 mice (both male and female) were used. All experimental mice were free of pathogens and were on a 12-12 hour light-dark cycle, with unlimited access to water and food. Temperature in the facility was controlled and maintained at 21 oC.
Wild animals	The study did not involve wild animals.
Field-collected samples	The study did not involve samples collected from the field.
Ethics oversight	All experiments involving mice have been regulated by the Animals (Scientific Procedures) Act 1986 Amendment Regulations 2012 and additional ethical review by the University of Cambridge Animal Welfare and Ethical Review Body (AWERB). Experiments were authorised by the Home Office (Licence number: 70/8864).

Note that full information on the approval of the study protocol must also be provided in the manuscript.

Flow Cytometry

Plots

Confirm that:

- [x] The axis labels state the marker and fluorochrome used (e.g. CD4-FITC).
- [x] The axis scales are clearly visible. Include numbers along axes only for bottom left plot of group (a 'group' is an analysis of identical markers).
- [x] All plots are contour plots with outliers or pseudocolor plots.
- [x] A numerical value for number of cells or percentage (with statistics) is provided.

Methodology

Sample preparation	As described in the Method, ES cells cultured on gelatin-coated plates were trypsinized to generate single cells. TS cells cultured on MEFs were trypsinized into single cells and then plated onto gelatin coated plates for 30 min to eliminate MEFs.

Single ES and TS cell suspensions were collected, fixed in 4% PFA, and permeabilized for 30 min at room temperature using 0.3% Triton-X-100 and 0.1% glycine. Cells were then incubated with anti E- or P-cadherin antibody for overnight incubation at 4°C in blocking buffer (PBST containing 10% FBS). Cells were washed twice in PBST and then incubated with secondary antibody (1:500 dilution) in blocking buffer at room temperature for 1-2h.

Instrument

flow cytometry (BD Biosciences)

Software

FlowJo software (https://www.flowjo.com)

Cell population abundance

We performed FACS experiment to identify the intensity distribution of E- and P-cadherin, no cell population were sorted in this work

Gating strategy

1.) SSC vs. FSC gating to exclude debris. 2.) FSC-H vs. FSC-A gating to exclude doublets.

☒ Tick this box to confirm that a figure exemplifying the gating strategy is provided in the Supplementary Information.
